# Aging in place: implementation facilitators, barriers, and strategies in population health-oriented active aging centers in Singapore

**DOI:** 10.3389/fpubh.2026.1748178

**Published:** 2026-05-04

**Authors:** Yan Zhi Chan, Sungwon Yoon, Audrey Shu Ting Kwan, Lian Leng Low

**Affiliations:** 1 Duke-NUS Medical School, Singapore, Singapore; 2 Health Services Research and Population Health, Duke-NUS Medical School, Singapore, Singapore; 3 Centre for Population Health Research and Implementation, Singhealth, Singapore, Singapore; 4 Division of Population Health and Integrated Care, Singapore General Hospital, Singapore, Singapore; 5 Department of Physiotherapy, Singapore General Hospital, Singapore, Singapore; 6 Research and Translational Innovation, Singhealth Community Hospitals, Singapore, Singapore; 7 Singhealth Duke-NUS Family Medicine Academic Clinical Program, Singapore, Singapore

**Keywords:** active aging centers, aging, aging-in-place, implementation research, older adults, population health

## Abstract

**Background:**

As populations age rapidly, community-based care is crucial to support aging-in-place. Yet, few models successfully integrate health promotion, social engagement and care coordination at scale. Singapore’s Active Aging Centers (AACs) represent a novel, nationally coordinated and funded model that integrates health promotion, social engagement, and care coordination for older adults. Despite the increasing policy importance, little is known how such models are implemented and sustained in practice.

**Objective:**

This study examined the facilitators and barriers of implementation of AACs from the perspective of frontline staff.

**Methods:**

We conducted a qualitative study guided by the Consolidated Framework for Implementation Research (CFIR). Semi-structured interviews were performed with 21 center managers and staff in Singapore. Participants were purposively sampled to reflect diversity in operating models and local demographics. Transcripts were analyzed using an inductive-deductive approach, and themes were mapped to CFIR domains.

**Results:**

Participants viewed the AAC model as conceptually strong and well-aligned with aging-in-place goals. Key facilitators included holistic policy design and community partnerships. Barriers included resource constraints, difficulty engaging underrepresented senior groups and a top-down approach in implementation. Findings also highlight implementation dilemmas of control against frontline flexibility. Novel strategies such as the thematic approach, senior volunteers, and culturally-tailored outreach were adopted to improve engagement and sustainability.

**Conclusion:**

Singapore’s AAC model demonstrates promise as a population health–oriented, community-based aging intervention. Effective implementation requires flexibility in policy and closer alignment between frontline experiences and central governance. These insights can guide policymakers and aging-in-place systems globally and across the region aiming to build resilient, integrated community care ecosystems.

## 1 Introduction

The global population is aging rapidly (1). According to the World Health Organization (WHO), by 2030, one in six people worldwide will be over the age of 60 (2, 3). This demographic shift presents major challenges for health systems due to the burden of chronic conditions, many of which are influenced by modifiable lifestyle and environmental factors (4). In response, there is growing emphasis on preventative and community-based interventions that support healthy aging (5, 6). This is aligned with WHO’s vision in its “Decade of healthy aging” for age-friendly communities, person-centered integrated care and community-based social care (7, 8).

Providing care for seniors in the community is a key policy and implementation challenge, especially in ensuring continuity of care (9, 10). Seniors with high care needs require substantial resources—doctors, nurses, social workers, allied health professionals in a multi-disciplinary team for each senior (11). With limited resources, many healthcare systems struggle to meet these demands (12). This underscores a critical need for aging societies: how can we implement health and community care for senior population with “lower care needs” to keep them active and healthy thereby preventing progression to higher care needs?

Singapore is experiencing rapid population aging: by 2030, one in four Singaporeans will be aged 65 and above. In response, the government has launched AgeWell SG, a national strategy which coordinates efforts across housing, transport, active aging and care services to support seniors in aging confidently within their communities. At the heart of this approach are Active Aging Centers (AACs) which serve as one-stop hubs for health interventions and promotion, social engagement, and care navigation.

While AAC-like centers or systems exist globally, their structures, funding mechanisms, and roles vary widely (13–18). Similar systems or centers which focus on seniors with lower care needs exist in countries such as Australia (Commonwealth home support program—CHSP) (19–22), Japan (Integrated Care Systems—ICS) (23–25), Canada (Senior Centers) (13, 26) and South Korea (Senior Welfare Centers) (27, 28), which aim to provide social and preventive services, support independent living and meet daily and health needs (29–35). The aims of these programs differ in their primary aim. The Candian and South Korean centers primarily aim to provide seniors with social support while the Japanese and Australian programs are home-based and focus on providing seniors with healthcare assistance. The Singapore “AAC model,” on the other hand, provides both social support and healthcare assistance. In terms of the funding model, the AACs in Singapore are centrally governed and funded at the national level with the closest similarities seen in the model from South Korea (36–39), and adopt a population health focus. This model, which providing both social support and with a healthcare aim, is novel, and its implementation is not widely understood (18). Existing studies tend to focus on the perspectives of older adults or high-level policymakers (14–17, 28), with limited attention paid to the insights of frontline staff. The perspectives of these frontline workers are crucial, especially in centrally governed policies, as they provide insights into how these policies translate into real-world settings as well as key implementation determinants and adaptations on the ground.

This study aimed to bridge this gap by examining the implementation of Singapore’s AAC model using the Consolidated Framework for Implementation Research (CFIR). The primary aim of the study was to identify barriers and facilitators in the implementation of AACs from the perspectives of frontline staff with focus on organizational, policy and contextual factors that influence implementation. The secondary aim was to explore strategies used by centers to overcome barriers and build on their strengths. By exploring frontline experiences within a CFIR-infromed analysis, this research provides contextually grounded in sights into the implementation of community-based aging interventions, with potential relevance for similar health-social care models in other settings.

## 2 Materials and methods

### 2.1 Study setting: “AAC model” and context in Singapore

Active Aging Centers (AACs) in Singapore is a relatively new community-care model introduced in 2021. Designed as one-stop drop-in centers for seniors above 60 years of age, AACs aim to cater to the health and social needs of these seniors. The “AAC model” was developed as an improvement to the prior Senior Activity Center (SAC), which focused on providing seniors with recreational activities. In comparison, AACs’ roles were expanded beyond social engagement to include more proactive health and social care functions. Staff members are required to outreach to identify seniors who may require assistance in the community. AACs’ functions are conceptualized in the “ABC + 2S” framework. As shown in Table 1, these include delivering Active Aging programs (A), conducting befriending and outreach to socially isolated seniors(B), facilitating care referrals to health and social services(C), supporting community health screening (S1), and acting as social connectors with primary care providers (S2). Together, these expanded functions position AACs at the forefront of engaging seniors living alone, enabling early identification of health and social needs, and serving as a key bridge between the community and primary healthcare system.

**Table 1:** ABC + 2S components that each AAC is expected to deliver.

Function	Description
A (“Active Aging”)	Conducting Active Aging Programs (AAPs) which are activities for seniors to participate and keep active
B (“Befriending and buddying”)	Reaching out and regular check-ins with the needy seniors at risk of social isolation
C (“Care Referral”)	Providing seniors with information on schemes or grants and providing seniors with referral to health and social services that they may require
S1 (“Community Screening”)	Ensuring that the health status of seniors is assessed
S2 (“Social Connector”)	Primary care providers are expected to refer seniors to AACs with a care plan

**Figure 1:**
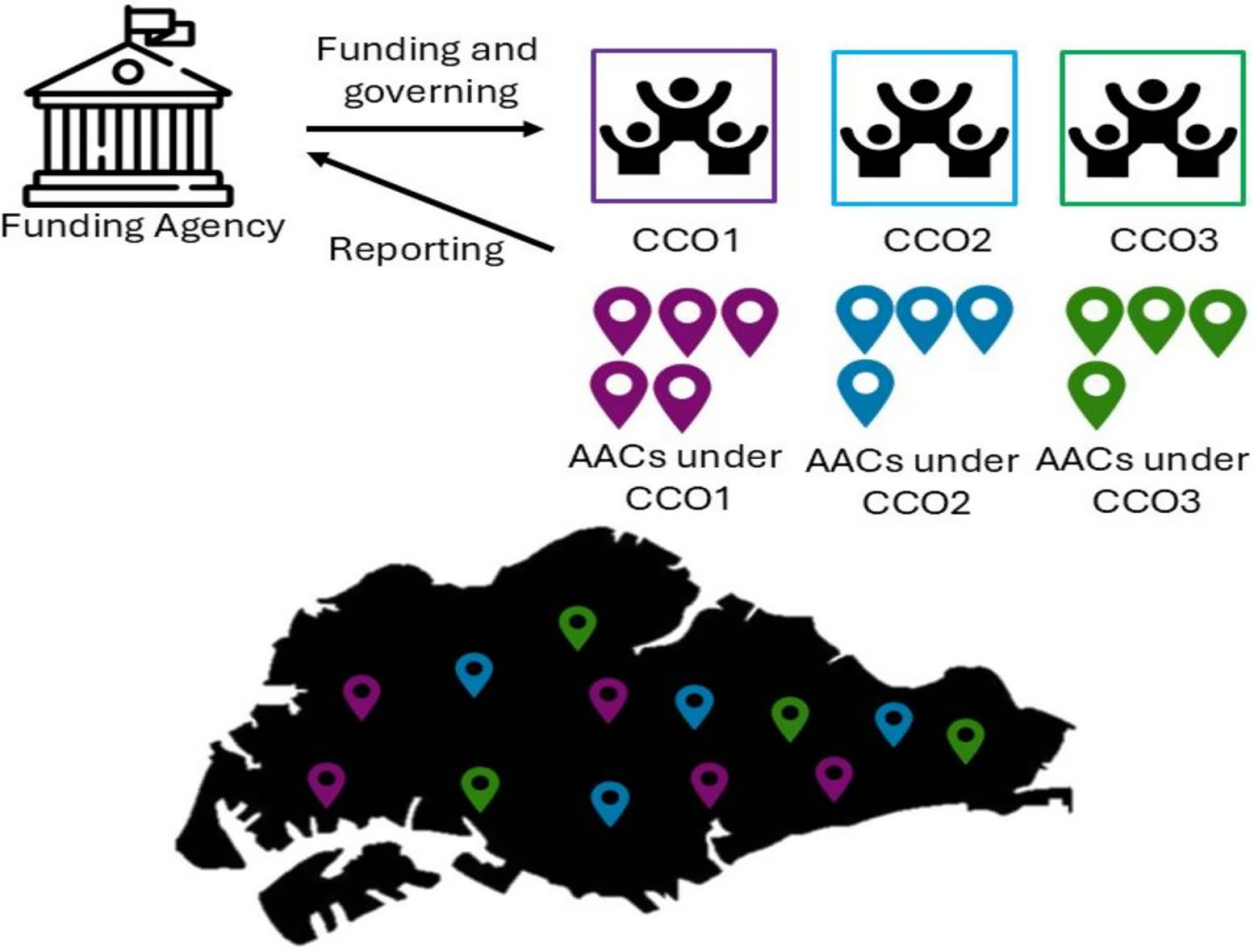
Funding and governance structure of AACs in Singapore.

As compared to community-care models in other countries, the “AAC model” is unique in its governance, funding and policy implementation. As shown in Figure 1, the AACs are funded by a funding agency that supports their operation. Most of the AACs are delivered at the ground level by center managers and staff, and may operate either as standalone centers or under the support of a Community Care Organization (CCO). CCO-supported AACs are overseen by a parent organization that manages multiple AACs across the country, providing administrative support, shared resources and leadership guidance. In contrast, standalone AACs operate more independently. At the time of writing, there are over 220 AACs across Singapore (40).

Each AAC is assigned a geographical boundary and a corresponding “target population” of older adults living in that region. Each AAC is accountable for a set of key performance indicators (KPIs), including the number of seniors engaged by staff members and program participation rates. Which are reported to the funding agency. Collectively, this performance and accountability structure supports a population health approach.

### 2.2 Consolidated framework for implementation research

Implementation science frameworks have been increasingly popular in health sectors due to its ability to investigate how policies and interventions can be applied in various contexts (41). The Consolidated Framework for Implementation Research (CFIR) is a determinant framework that is useful in determining the facilitators and barriers for intervention implementation (42). CFIR comprises five domains (Intervention Characteristics, Characteristics of Individuals, Inner Setting, Outer Setting and Process) and 39 constructs and subconstructs, providing a comprehensive structure for assessing implementation determinants. CFIR was selected for its comprehensive, multi-level approach, enabling systematic analysis of policy, organizational, and contextual factors influencing implementation. Its domains are well suited to complex, policy-driven community aging interventions like AACs, which require coordination across agencies, organizations, and frontline staff. The CFIR-ERIC matching tool was created by a group of implementation science experts and published by Powell et al. The tool links CFIR contracts to implementation strategies that can be used to address identified determinants. In this study, the CFIR framework was applied in two ways. Firstly, the CFIR framework and its domains and subdomains were used to develop the semi-structured interview guide to support holistic exploration of stakeholders’ perspectives on the “AAC model.” Secondly, the CFIR framework guided the analysis by providing a framework for mapping and organizing the derived codes into relevant domains and constructs.

For the secondary objective of the study, the strategies used to address the identified determinants (facilitators and barriers) were extracted from the data and mapped onto the CFIR-ERIC matching tool to identify the associated implementation strategies (43, 44).

### 2.3 Study design

The study adopted a qualitative approach that involved one-to-one in-person semi-structured interviews with AAC center managers and staff members.

This study was approved by the SingHealth Centralized institutional review board (Reference number: ECOS 2017–2,597).

### 2.4 Sampling

The eligibility criteria included individuals who were, at the time of the interview, a center manager or a staff working at the AAC and able to converse in English or in Chinese. All interviews were conducted between October 2024 and January 2025. Participants were recruited using purposive sampling to ensure a diverse representation of center managers and staff from a variety of CCOs and from a diverse range of public and private housing flats served (e.g., rental flats, 3-room flats etc.).

After initial pilot interviews, it was noted that there were mainly two types of AACs—one which is supported by CCOs experienced in running AACs and others which functioned more as a “standalone” center. This was also used as a metric to recruit participants for the interview to ensure that both groups were well represented in the study.

### 2.5 Data collection

A semi-structured interview guide was developed based on literature review (14–17) and the CFIR domains (42). All AACs in the Southern and Central region of Singapore were identified and some were purposively sampled based on characteristics such as CCO type and type of public housing served. Once identified, the center managers at the AACs were approached by email and invited to participate in an interview. Those who agreed had a convenient time arranged for the interview.

The interview guide was pilot tested with 3 participants (data were included). All interviews were conducted in person in English or Mandarin by one of the study team members trained in qualitative interviews (YZC). Participants were informed of the study’s aim and written consent was obtained. Each interview lasted approximately 90 min in duration in a private setting within each AAC. Field notes were taken during the interviews.

### 2.6 Data analysis

All interviews were audio recorded, de-identified and transcribed verbatim. All transcripts were verified by the study team to ensure accuracy. Interviews conducted in Mandarin were transcribed verbatim and translated into English, with translations checked by members of the study team to ensure accuracy and consistency. Data analysis was performed in the following steps: (1) familiarization of the data by reading the transcripts line-by-line and listening to the original recording of the interviews, (2) coding of the data by categorizing transcript segments into meaningful groups. This process was mapped onto the constructs and subconstructs of the CFIR framework, which provided a structured lens for analyzing the data. Three study team members (YZC, KSTA and SY) were involved in the process. Initially, two coders (YZC, KSTA) independently coded the transcripts in an inductive-deductive fashion, with the guidance of the CFIR framework, and discrepancies on a coding frame were resolved through iterative discussions involving the third study member (SY). Agreement was reached among the team members on the coding frames, constructs and overarching themes regarding key factors influencing the implementation and strategies to address them. Data saturation was assessed across the CFIR domains. Saturation was considered achieved when no new themes emerged and all domains were adequately represented in the data. Coding consistency was ensured through this team-based consensus process rather than formal inter-coder reliability statistics (e.g., Cohen’s kappa). NVivo 14 software facilitated the analysis process for categorizing codes and themes under each CFIR constructs and subconstructs and for management and organization of the data. An audit trail was maintained throughout the study to document key decisions during data collection and coding process. Reflexivity was considered through regular team discussions on researchers’ roles and potential influence on data interpretation. An iterative approach was adopted, with early findings informing refinements to the interview guide. Data collection and analysis conducted concurrently to allow continuous comparison and refinement of themes.

## 3 Results

### 3.1 Participant characteristics

A total of 21 participants were interviewed through 20 sessions. One interview session included two participants due to time constraints, with the rest of the interviews conducted one-to-one. Table 2 shows the demographic characteristics of the participants. As noted in section 2.4, there were 2 types of centers—ones which were standalone and those that were more CCO-supported—the numbers of each shown in Table 2.

**Table 2:** Participant characteristics (*n* = 21).

Participant characteristics	n (%)
Age (years), mean (range)	44.6 (22–65)
Sex	
Male	8 (38%)
Female	13 (62%)
Ethnicity	
Chinese	18 (86%)
Others	3 (14%)
Position	
Center managers or equivalent	12 (57%)
Staff	9 (43%)
AAC type	
Standalone AACs	6 (28.6%)
CCO supported	15 (71.4%)

### 3.2 Facilitators and barriers to implementing the “AAC model”

**Figure 2:**
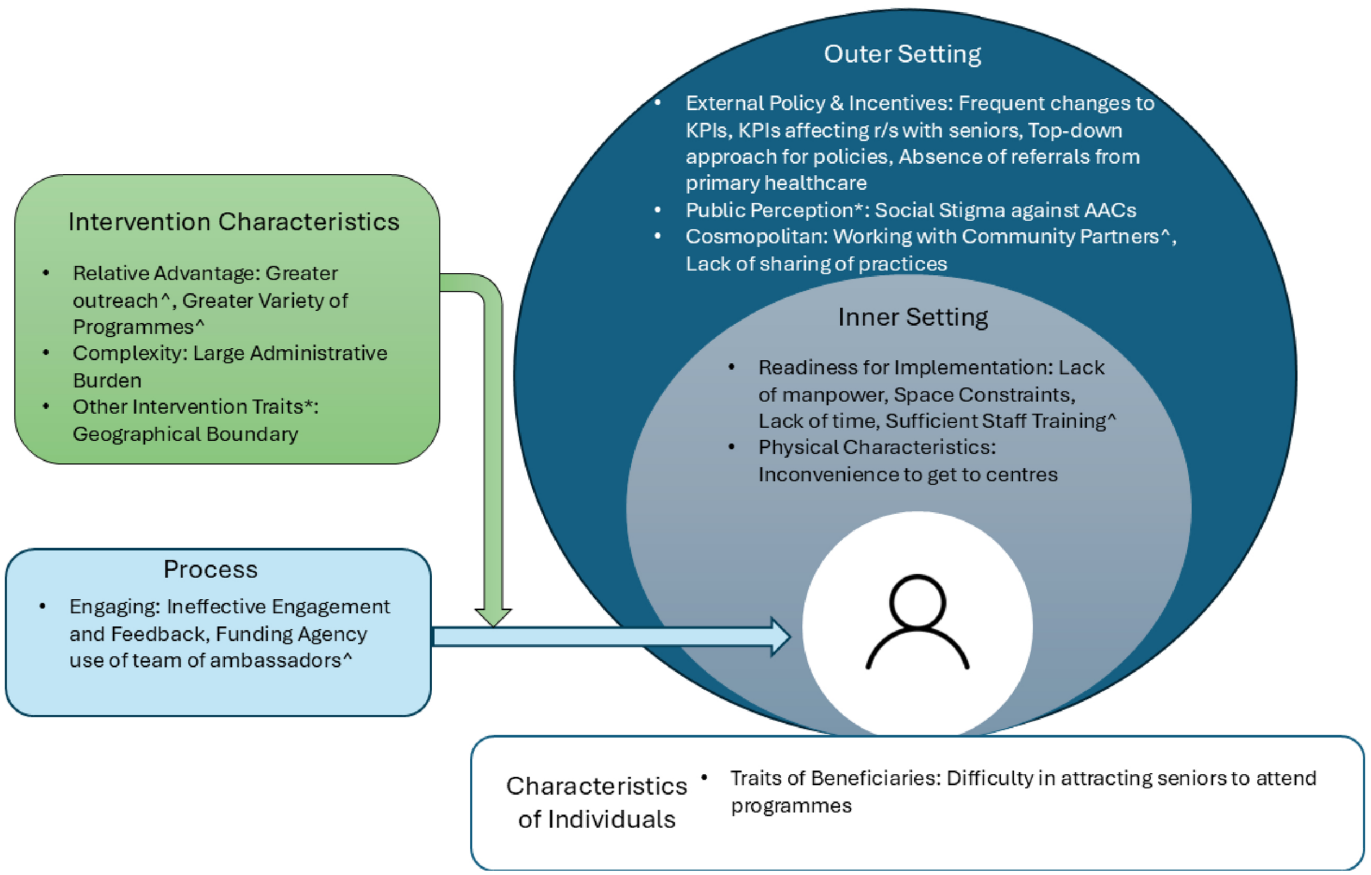
Key facilitators and barriers to AAC model implementation in CFIR domains and constructs (*means open-coded construct, ^means facilitators).

Findings are presented using the CFIR domains. Supplementary Table S1 presents the comprehensive domains, constructs, subthemes and representative quotes while Figure 2 provides a visual overview of the results. The key CFIR construct most frequently discussed was the outer setting. This reflects the critical role of community partners and the funding agency in implementing AACs on the ground. Additionally, this area represented the most significant change from the prior SAC model and, therefore, offers the greatest opportunity for improvement.

#### 3.2.1 Intervention characteristics

The intervention characteristics domain describes the “AAC model” and its traits that may influence its effectiveness in implementation on the ground.

Participants viewed that the “AAC model” was appropriate to provide all-round care for the seniors, citing an increased outreach to seniors and increased variety of programs as two key benefits. However, barriers included an increasing operational complexity, challenging targets set by the funding agency and limited funding allocated to run programs. This identifies a key conflict between ease of implementation and variety of programs provided for seniors.

*“I also see that it [“AAC model”] forced us to reach out to more. Okay but I feel, it will make you not serve the same seniors again and again. You will go and reach out to these[new] seniors…” #16, Centre Manager*

*“…Oh my god, the amount of administrative work that we have to do. Because for outreach, you need to key in the data, and the amount of data that you need to key in is a lot. When it comes to reporting, you need to do [funding agency]‘s reporting. Some more there isn’t just one report, there are so many reports.” #9, Centre Manager*

A key structural concern by participants was the assignment of geographic service boundaries. AACs are only accountable for serving seniors within their designated boundaries, and services provided to those living outside these boundaries are not counted toward performance targets. This constraint limited flexibility and responsiveness, despite most AACs’ willingness to serve all seniors.

*“Because… … if they [funding agency] set the boundary here, and you actually let out-of-boundary seniors come, then it defeats the purpose because it did not hit your KPI, that they told you.” #14, Staff*

#### 3.2.2 Characteristics of individuals

The “characteristics of individuals” domain explores the characteristics of seniors, center managers and staff that affect “AAC model” implementation.

Many AACs found it difficult to engage certain subgroups of seniors. These includes ethnic minorities, males, socially isolated, “younger” seniors—seniors who are financially stable, with a higher socioeconomic status and technologically savvy—and those with competing commitments (e.g., employment or caregiving responsibilities). The main reason gathered was that many felt out of place in a center dominated by older Chinese females. A pertinent concern was the failure to attract “young” seniors. Traditional AAC offerings, such as karaoke, simple exercises, or board games, often fail to appeal to this emerging demographic which questions the sustainability of the “AAC model.”

*“If you’re in a minority group, and you go in, then ten people there, nine of them are all Chinese. Then let's say I'm an Indian. … … you feel like you're the odd one out.” #5, Centre Manager*

*“Because we also must see the changing needs of the seniors in Singapore, the kind of demographic of seniors in Singapore. Because the current AAC model, I don’t know if it’s very appealing to, let’s say, (in) their early 60s…So, I some things would need to change.” #10, Centre Manager*

#### 3.2.3 Inner setting

The inner setting domain explores the internal organizational context of the AACs and the CCOs, including infrastructure, staffing and culture.

Limited manpower, space and time were consistently cited as barriers. Many participants perceived that the limited manpower, coupled with high center workload makes it difficult to complete their day-to-day tasks, affecting their ability to serve existing seniors and reach out to more. Similarly, limited spaces make it difficult to enable seniors to participate in activities.

*“I mean, of course, three [manpower] are already not a lot. So now short (of) one is tough. We work on very lean resources, three head counts including myself.” #11, Centre Manager*

*“…in the morning, sometimes you see the whole bunch of seniors outside, because in here is so small, we cannot fit so many [senior]. But when we turn them away, that means they may never come back.” #1, Centre Manager*

Many participants also recognized the training efforts for staff, both by the funding agency and by their individual CCO, in equipping them with the skills necessary for AAC operations.

*“For anyone who joins, it’s [training] helpful because it helps you with operations. Training is great also because, it just equips you with the kind of skills that you require at an AAC.” #10, Centre Manager*

Physical characteristics formed as an open-coded construct as the physical space of the AAC was noted to play an important role. Many staff highlighted the inconvenience in reaching the center due to long travel distances and physical barriers such as stairs or large roads, given the frailty of some seniors.

*“Because the way to our centre is that we are not in the center of the whole thing, and we are at the far end of the whole area we’ve been given. From one end to the other it takes a three-bus stop ride.” #1, Centre Manager*

#### 3.2.4 Outer setting

The outer setting domain explores external influences, including funding agency, policy and community partnerships.

A common frustration was the frequent changes in policy and administrative requirements, on the backdrop of increasing operational complexities. These were perceived as top-down with limited understanding of operational realities and the context on the ground. Participants reported unrealistic targets, vague implementation guidance and insufficient support from the funding agency.

*“Sometimes it [KPIs] changes quite rapidly….and it’s not that easy on us because as operators for AACs, it takes time to do some implementation or through some transition or change… the KPIs need to be really on the ground up. I feel it’s top down, but maybe [funding agency] people should come to the ground, work with the AAC people, understand the nature, the difficulty, the challenges before they build the policy and regulations on KPI upwards” #7, Staff*

A prevailing concern was the increasing focus of the funding agency on KPIs, which many perceived to shift the focus away from meaningful relationship building with seniors. While most participants agree that accountability is required, the excessive focus on numbers and targets was viewed as misaligned with the ‘holistic care goals’ of the AAC model.

*“It pushes us towards transactional relationship building, because you need to keep on hitting numbers, your focus is there, instead of that social capital building that you need to hold dear. Because old folks don’t trust that easy right?” #2, Centre Manager*

Another concern was the social stigma against AACs. This is on the background of public perception that AACs are focused on helping the financially and socially needy seniors instead of being for every senior. Thie has limited the appeal of AACs for many seniors.

*“Those who are well and healthy, able, be it financial, be it mental, be it physical, perhaps they don’t see the point to come down. “I’m well”. And those who are well, they feel like maybe I have my own clique. Our branding has been stereotyped under the low-income group” #3, Centre Manager*

A key facilitator for the effective running of AACs was the collaboration with other community resources like community centers, residential committees. This enables AACs to refer seniors to these other community resources and vice versa. However, some participants indicated that there is not much interaction between AACs unless they are within the same CCO, with little sharing of best practices between AACs.

*“I do work very closely with other community partners like our RC (residential committees), CC (community centres) and then [name of kindergarten].” #11, Centre Manager*

*“I mean I would like to know (how the nearby AACs are coping) … [It’s] good to know where we are standing in terms of having a yardstick to know how my AAC is doing. Right now, I don’t know how my surrounding AAC are coping.” #9, Centre Manager*

Despite this, many AAC frontline workers reported limited connection and referrals between AACs and primary care practitioners, despite this being an improvement in the “AAC model” as compared to the prior SAC model.

*“We never had referrals from there, from GPs (general practitioners) yet. … I think that the referral from the GPs is low because it’s not their focus. None of the referrals came through.” #9, Centre Manager*

#### 3.2.5 Process

This process domain reflects how the implementation process of the “AAC model” was experienced by participants.

Participants appreciated the team of ambassadors deployed by the funding agency who provided direct support and liaison. However, some participants felt that their feedback was poorly handled with little evidence that it resulted in meaningful policy or procedural changes. Participants felt there should be more constructive engagement with ground staff and support from the funding agency.

*“With the [name of team] team, I think we have closed the gaps. So, for anything we just go to our [name of team] who works very closely with us on the ground. I think that’s a good thing, appreciative about that.” #11, Centre Manager*

*“We will try to feedback to HQ [headquarters] and see what HQ can feedback to [funding agency]. And certain things, I think, sometimes, [funding agency] also do understand, they do ask our feedback, but we tell them the feedback, but they never come back with our feedback.” #7, Staff*

### 3.3 Strategies adopted by AACs

We explored strategies AACs used to navigate implementation challenges or leverage facilitators. Selected strategies employed by AACs are presented in Table 3 while a comprehensive list of strategies is presented in Supplementary Table S2. The strategies highlighted in this section were selected for their analytic relevance to the study objectives and for illustrating how implementation barriers were addressed in practice.

**Table 3:** Implementation strategies to address challenges.

Strategy employed	Challenges and facilitators (& CFIR construct)	CFIR-ERIC strategy	Brief description
Culturally appropriate programs to address individual barriers	Difficulty in engaging groups of seniors such as male seniors, ethnic minorities, “younger seniors” (Characteristics of individuals)	Promote Adaptability, Tailor Strategies	Targeted programs which aim to target difficult-to-engage groups
Novel “AAC branding and implementation”	Social Stigma against AACs (Outer Setting)	NIL	Redesigning individual AACs to have their “unique selling point”
Optimizing manpower and space resources	Limited Resource of AACs such as manpower and space (Inner setting)	Prepare consumers to be active	Resourcefully optimizing available resources to assist in AAC operations
Leveraging community networks	Working with other community resources (Outer Setting)	Build a Coalition	Increasing the interaction between AACs and other resources to provide all-rounded care for seniors and for better planning of activities

#### 3.3.1 Culturally appropriate programs to address individual barriers

AACs attempted to design programs to improve the engagement among underrepresented seniors such as males, ethnic minorities and “younger” seniors (CFIR: Characteristics of individuals). Initial approaches included gender-targeted activities like male-only badminton or tea appreciation sessions. These activities often saw limited uptake from male participants or were attended by female participants. This was attributed to preconceived notions of the demographics and personalities of a typical “AAC participant”. Many believe that they do not fit these perceptions, making it unlikely for them to participate despite the presence of “special programs” that target them. More effective approaches included informal drop-in spaces such as “transforming” AACs into coffeeshops which encouraged casual bonding, using Singapore’s coffeeshop culture to connect to male seniors. Similarly, collaborations with religious institutions increased ethnic minority participation by providing culturally familiar and comfortable settings. These strategies highlight the importance of tailoring programs to fit the everyday practices and preferences of seniors, consistent with CFIR-ERIC strategies on adaptation and tailoring.

*“Then we have all male drink coffee thing. So, we have that once a month only, a lot of places (other AACs) have it every week, but we have it once a month because ours was 30 over percent engagement of male seniors. They come here, they’re very happy...” #1, Centre Manager.*

#### 3.3.2 Novel AAC branding and implementation

AACs are sometimes associated with a stereotypical set of activities such as bingo, Rummy-o or mahjong which may contribute to a perceived stigma. In response, several AACs have sought to reimagine and reinvent how an AAC should be like. A unique approach was the adoption of a “thematic approach” for AACs. In this approach, each individual AAC has a specialized theme with facilities, activities and even the physical buildup of the center aligned with this theme. This enables interested seniors to participate in thematic activities catered to their interests, beyond the traditional AAC offerings. Another method to provide novel activities for seniors includes the facilitation of interest groups from the ground-up, enabling seniors to initiate and participate in less conventional activities. These strategies aim to address the negative perceptions of AACs (CFIR: Outer Setting), by moving away from a ‘one-size-fit-all’ model and creating more vibrant and engaging environment for seniors.

*“You want to make it like a club style that “wow, your centre is like that.” So, we are really looking into the thematic, as in the programmes that we run which is different. Like you see our [thematic programme], that’s something very unique.” #18, Centre Manager*

#### 3.3.3 Optimising manpower and space resources

To address manpower shortages, some AACs recruited and trained a core group of senior volunteers to assist with befriending, facilitating sessions and even buddying new seniors. In some centers, volunteer-led programs have become self-sustaining, freeing up staff capacity and improving sustainability of the AAC operations. For example, the “micro-jobber” scheme, reimburses seniors for completing simple tasks. This doubles up by empowering seniors to take an active role in AAC operations while addressing staff needs. Similarly, to address the space constraints within each AAC, many AACs used community resources and public spaces for larger activities. Access to such underutilized spaces enabled some AACs to organize activities that would otherwise be limited by physical capacity. Overall, these strategies address key resource constraints, particularly manpower and space (CFIR: Inner Setting). The micro-jobber scheme aligns with CFIR-ERIC strategies related to engaging consumers as active participants, as seniors contribute directly to AAC operations.

*“We have this programme called microjobbers. So, we empower our seniors to take on mini jobs, to which we will give them a small remuneration, but also ease the load on our AAC operations, as well as giving them a purpose when they are running their own activities..” #10, Centre Manager*

#### 3.3.4 Leveraging community networks

External partnerships, especially with other community resources, were seen as a critical enabler (CFIR: Outer setting). For example, collaborations initiated by local political leaders enhanced access to community resources and facilitated service integration. These relationships enabled AACs to offer more comprehensive and tailored services to meet seniors’ needs. Working as part of a broader network also allowed resources to be pooled and coordinated, enhancing collective capacity and supporting centers in achieving shared goals. This mechanism aligns with the CFIR-ERIC strategy of building a coalition, where community partners work together to better serve the seniors.

*“Of course, we are seated in the [name of intervention] …So she [political figure] brings all the community partners together and she wanted to deconflict a lot of the services and programs by the different partners but for the same group of seniors in her constituency. This is pretty unique and that’s the efforts of [political figure] at that level to bring the partners together. “#9, Centre Manager*

## 4 Discussion

This study identified key facilitators and barriers to implementing the population health-oriented and community-based AAC model in Singapore. It also highlighted strategies that AACs employed to address challenges. The analysis revealed five key areas influencing implementation: the design of the AAC model, challenges in engaging seniors, resource constraints, top-down policy implementation, and collaboration with community partners.

Participants generally viewed the AAC model as conceptually sound and well-aligned with the goal of supporting seniors to age in place (45, 46). Its emphasis on holistic, integrated care and increased outreach was seen as aligned with these care goals. In particular, active outreach to seniors living alone and screening for common chronic conditions were perceived to greatly enhance engagement, especially among those who may not otherwise access AAC services. The diverse, population health-oriented programs also allowed for participation across diverse groups of seniors. However, these strengths were accompanied by concerns about the increasing complexity in program delivery and administrative requirements as the model matures. Many centers found these demands burdensome given existing resource constraints, highlighting an inherent tension between operational feasibility and the pursuit of more comprehensive care goals.

One of the most significant implementation challenges was engaging seniors to participate in AAC programs. Consistent with findings in both local and international literature, AACs struggled to attract specific subgroups (13, 14, 33). In particular, “younger seniors” underscores were less interested in traditional offerings such as Bingo, highlighting a mismatch between the current “AAC” program design and evolving aging trajectories (47). Innovations such as “thematic AACs” represent a positive step, though these interventions came at an increased cost and resources. The “lim kopi” program was an example of a culturally resonant initiative which mimics the coffeeshop culture in Singapore, successfully attracting male seniors (48). Similarly, partnerships with religious institutions yielded an increase in ethnic minority participation as these seniors were more comfortable with the involvement of religious institutions. Together with other successful ground-up innovations such as the “micro-jobber” scheme, these examples underscore the importance of leveraging cultural context, frontline feedback, and local initiatives and collaboration. The primary limitation remains the capacity to rapidly scale these innovations through policy channels.

Compounding the challenges of senior engagement were significant constraints in manpower, space, and time. With limited funding and persistent staff shortages—exacerbated by low salaries—many AAC teams were overstretched. These challenges echo issues faced in senior care models in other countries (15, 49, 50). One mitigation strategy has been to empower seniors to take on roles within the AACs, such as facilitators, befrienders, or administrative assistants, relieving staff workload and promoting a sense of purpose and community ownership among seniors (51, 52). Further support, such as the introduction of “micro-jobber” schemes in selected AACs could incentivize and sustain this model, making AACs more resilient and self-sufficient over time (53, 54). Scaling up such proven schemes across all AACs would provide broad benefits nationwide and offer a valuable lesson for other community-based health systems regionally and globally. By empowering seniors to contribute, these initiatives can reduce resource strain while enhancing their sense of purpose, a win-win situation for all.

Participants also described significant implementation challenges arising from top-down policy implementation. Limited ground-level understanding by funding agencies led to unclear guidance, frequent policy shifts, and unrealistic targets. These contributed to a growing sense of misalignment between AACs’ core mission and their administrative obligations. While the presence of liaison teams from funding agencies was welcomed, participants expressed frustration that their feedback did not result in substantive policy changes. Moreover, the emphasis on outreach KPIs has altered the nature of staff–senior relationships, rendering interactions more transactional and undermining the trust and rapport between seniors and staff. Frontline staff are incentivized to recruit as many new seniors as possible, often at the expense of spending time building relationships with existing participants. This disconnect could dilute the AAC’s mission, staff morale, and long-term sustainability. The unique central funding and governance structure of the “AAC model” may likely contribute to this outcome through its top-down implementation. While this approach offers benefits such as greater control over equity and distribution of funds and resources, it also means that the model is implemented with a “big picture” view, often at the expense of ground-level experiences. Compared to alternatives such as trust-based or relational approaches, the current KPI model still helps maintain accountability and ensures alignment of realities on the ground with higher-level targets. Technology can play a key role in bridging the gap between frontline staff and policymakers by enabling two-way communication to facilitate more responsive policy development, and ensuring proper alignment on the ground. Overall, more responsive policymaking that leverages technology and is grounded in frontline experience, is essential to balance both accountability and relational impact (39, 55, 56).

The challenges in the top-down policy implementation and the “innovation” and adaptability reveal a key implementation tension between control given to the funding agency and flexibility given to ground staff. While oversight by the funding agency enables standardization, accountability, and equitable program reach, it also shapes implementation behavior through performance-driven expectations that may constrain local responsiveness. In contrast, flexibility at the level of frontline staff and their respective CCOs creates space for contextual adaptation, relational engagement, and responsiveness to seniors’ needs. This tension reflects a broader dynamic in implementation science, in which the imperatives of scalability and control must be continually balanced against the need for flexibility and innovation on the ground. In the case of AACs, the idea of the KPIs was intended to provide a balance between control and flexibility. However, the bar for KPIs was set too high for participants to truly innovate and adapt, leading them to overly focus on achieving KPIs.

Collaboration with community resources was frequently cited as a facilitator of successful implementation. AACs, while vital to Singapore’s active aging strategy, often lack the capacity to meet complex health and social care needs of their senior clients. Recognizing these concerns, the Integrated Community Care Provider (ICCP) model has been implemented—a service delivery approach that integrates key community care services, including AACs, home personal care, day care, and home therapy, under a regional provider (57, 58). ICCPs are intended to serve as sub-regional hubs that coordinate long-term care through unified care plans and optimizing the use of community resources. Singapore’s three public Healthcare Clusters are responsible for leading ICCPs in aligning community-based health and social services with regional health strategies (59). Although some collaboration already exists within larger CCOs operating multiple AACs, strengthening cross-agency integration is key to enhancing implementation quality, promote service equity, and position AACs more effectively within the integrated care ecosystem (56, 60).

Finally, when analyzing the strategies employed by AACs, the CFIR-ERIC matching tool did not map the strategy “novel AAC branding and implementation” to any of the 73 implementation strategies. The CFIR-ERIC tool is designed to link identified facilitators and barriers to predefined implementation strategies, aimed at improving the uptake or effectiveness of specific interventions. In contrast, the AAC model for this study allows flexibility in how ground-teams achieve targets. This discrepancy suggests that additional implementation strategies beyond those captured by CFIR-ERIC may need to be considered.

All in all, Singapore benefits from its small size and high population density—allowing each AAC to cater to anywhere from 800 to 4,000 seniors living within a stone’s throw away. Some countries implementing home-based services, such as Australia and Japan, could consider a similar center-based strategy in densely populated urban areas which may be less resource-intensive than current home-based strategies. The “AAC model” could serve as a prototype for comprehensive senior care, integrating both social and healthcare services under one roof. Its centralized funding and governance structure enables more equitable and population-level benefits for seniors. Its implementation in the context of regional health systems could inform the design of similar centers in larger communities. However, even with its clear structure, there remains room for improvement—particularly by incorporating input from all key stakeholders, including frontline workers and even primary care. Co-creation could enable other similar center-based systems to address the shortcomings of the “AAC model.” Increasing primary care physician involvement could further close the loop, strengthening a model that may shape the future of integrated community care systems.

### 4.1 Limitations

This study has a few limitations. This study was conducted within the “AAC model,” and therefore, findings may not transfer directly to other health and social care systems, particularly in larger countries. In addition, as participation in the interview was voluntary, staff who were more open to sharing their perspectives may have been overrepresented, introducing potential self-selection bias. This study focused on frontline staff and did not include the perspectives of older adults, limiting triangulation of findings. Future research should integrate both provider and user perspectives to enhance the comprehensiveness of conclusions. Participants were recruited from Active Aging Centers in the Central and Southern regions of Singapore. While these regions encompass a diverse range of housing types and community settings, the study may not fully capture the diversity of challenges faced by AACs across the country. Nevertheless, efforts were made to reflect variation in AAC settings through purposive sampling and by including participants from different types of AACs. Similarly, it should be noted that 86% of the participants were ethnically Chinese and that a majority of participants were female. While this reflects Singapore’s national demographic distribution and the gender distribution in the community care workforce, it may nevertheless influence the perspectives captured in the study. Nevertheless, the findings provide valuable insights that may inform the adaptation of similar population health-focused aging-in-place models in other contexts.

## 5 Conclusion

This study highlighted critical enablers and challenges in implementing Singapore’s Active Aging Center model, with lessons applicable to aging societies regionally and globally. While the model is broadly aligned with older adults’ needs, successful implementation requires more than a standardized approach. Flexibility in policy design, stronger alignment of performance metrics with ground realities, and sustained support for local innovation are essential for effective implementation. As countries in the region expand community-based eldercare, these findings underscore the importance of empowering frontline implementers and tailoring interventions to diverse local contexts. Future work on AACs and their implementation could involve more in-depth evaluations of innovative practices, such as the thematic approach and ground-up adaptations like the “micro-jobber” scheme, to assess their impact. Operational improvements should also be examined especially the use of technology to enhance communication between ground staff and funding agency, as well as interactions with seniors. Such evaluations can provide valuable insights on how the “AAC model” can evolve to become more effective and sustainable for future aging populations.

## Data Availability

The original contributions presented in the study are included in the article/Supplementary material, further inquiries can be directed to the corresponding author.
