# Supplementary material for: Aging in place: implementation facilitators, barriers, and strategies in population health-oriented active aging centers in Singapore
Source: Front Public Health. 2026 May 4;14:1748178. doi: 10.3389/fpubh.2026.1748178 (PMC13180892; doi:10.3389/fpubh.2026.1748178)
Supplement: Supplementary file 1 [file Table_1.pdf]

Table S1. Barriers and facilitators for AAC model implementation according to the CFIR framework.

CFIR Domain	Construct	Definition/Meaning	Facilitators and barriers	Representative Quotes
Intervention Characteristics	Relative Advantage	Stakeholders' perception of the advantage of the "ABC+2S" model against the SAC model/transition model	Greater Outreach (F)	*"I also see that it ["AAC model"] forces us to reach out to more [seniors]. Okay but I feel, it will make you not serve the same seniors again and again. You will go and reach out to these [new] seniors…" (#16, CM)*<br>*"Because of this move, we were able to reach out to people within the community that (we weren't). We chance upon families that really don't know where to seek help and … … point them towards the right direction. " (#2, CM)*
			Greater variety of programmes (F)	*"Last time, … … the centre is not obliged to run all sorts of activities for them. Now, there are all these diverse things that they can look forward to. (#9, CM)*
	Complexity	Perceived difficulty in implementation of intervention	Difficulty in achieving targets (B)	*"Yeah, so it's a bit difficult in a sense, like KPI-wise. But I do understand why KPI is needed. It's just that people don't see how difficult it is to achieve them" (#19, S)*<br>*"So, we have a lot… … I think one of the greatest challenges of AAC is we have a lot of KPI to meet in a year…" (#12, S)*
			Large administrative burden (B)	*"…Oh my god, the amount of administrative work that we have to do. Because for outreach, you need to key in the data, and the amount of data that you need to key in is a lot. When it comes to reporting, you need to do [funding agency]'s reporting. Some more there isn't just one report, there are so many reports." (#9, CM)*
	Cost	Financial impact of the "AAC model" on AAC based on allocated funding provided	"Minimal" Funding (B)	*"And they are giving us funding that is barely sustainable" (#2, CM)*<br>*"But some… uhm… sometimes we are restricted by funding. Yeah, that's the thing" (#7, S)*

	Other intervention traits (open-coded)	Any other characteristics of the intervention.	Geographical Boundary limiting effectiveness (B)	*"So, if you look at it at a higher level, my AAC is taking care of seniors and their well-being, except that the target group is non-cluster [seniors who are not within boundary]. But I'm still doing my job, the resources that is put in, the time that is put in … But you know other than hitting target, we are also serving non-cluster. So will we be rewarded for that?" (#14, S)*<br>*"Because… if let's say they [funding agency] set the boundary here, and you actually let out-of-boundary seniors come, then it defeats the purpose because it did not hit your KPI that they told you." (#14, S)*
Characteristics of individuals	Traits of beneficiaries (open-coded)	Traits of seniors such as demographics, health status and other circumstances that directly or indirectly impact the successes of the intervention.	Difficulty in attracting seniors to attend programmes (Ethnic minority, males, "young" seniors, Language barrier, other commitments, worry of gossip, basic choice) (B)	*"If you're in a minority group, and you go in, then ten people there, nine of them are all Chinese. Then let's say I'm an Indian. … you feel like you're the odd one out." (#5, CM- minorities)*<br>*"Because we also must see the changing needs of the seniors in Singapore, the kind of demographic of seniors in Singapore. Because the current AAC model, I don't know if it's very appealing to, let's say, (in) their early 60s…So, I some things would need to change." (#10, CM- "young" seniors)*<br>*"Hard to come down. I would say generally all the uncles are more difficult. Not just for this centre but I would say generally." (#5, CM- male seniors)*
			Difficulty in getting seniors to centres (Socially isolated, Working seniors, seniors with mobility issues) (B)	*"My job is to make sure that those very frail and non-active seniors that actually come out from their house. So, the challenges are, [it is] really very hard to get them to come out." (#18, S-socially isolated)*<br>*"Okay then those who are like maybe socially very isolated, they don't want to mix around" (#20, S- socially isolated)*<br>*"when I do home visitation typically, I can't reach them (working seniors).... But perhaps I get gather some information from the*

				husband that she works. [She] come back [late in the evening and leaves early in the] morning so I cannot engage [her]… … we may want to categorize which senior [are] working or not because working one [seniors] it's very hard to reach out to them." (#14, S-working seniors)<br>*"And I guess a lot of them socially isolated is because of mobility issues, they cannot go out, or they cannot come to the centre." (#11, CM – mobility issues)*
			Difficulty in contacting seniors (scams, private properties) (B)	*"Because the thing about calls, the issue now is a lot of them are afraid of scam calls. So, that's another thing we'll fight with." (#10, CM- scams)*<br>*"Because about scams right a lot of times people don't pick up the call because it is an unfamiliar number." (#14, S- scams)*<br>*"But for condominium [private property in Singapore], where there's security, they just ask "what's the purpose?" They won't allow you to go in. So, there is a challenge to reach out to this group." (#2, CM- private housing)*
			Mindset of seniors (B)	*"To at least get them to experience the new thing. That is when we have difficulty." (#4, S-apprehension for new activities)*<br>*"But sometimes, they really feel like, I don't want to call entitled, but they can be… they have been so used to getting things all the time. And then maybe it's just like one time that they don't and then they start acting out." (#10, CM- entitled seniors)*
	Knowledge and Belief about the intervention	The attitudes of Centre Managers and staff of AACs towards the "ABC+2S" model	Belief that the idea of the intervention is good (F)	*"The concept is good, but it just needs to be moderated." (#2, CM)*<br>*"So, it is quite heartening to see some of these policies are implemented. I can see the concept and the theory behind what's being done." (#10, CM)*
	Other personal attributes	Personal traits of staff and centre managers such as motivation,	Staff express passion to serve (F)	*"Because the reason why I stepped into social service line, is to be able to serve, able to assist the seniors in particular, as much as I*

		values, competence and capacity.		*can." (#5, CM)*<br>*" I said something like… it has to be a calling. Working in an AAC right is physically tiring and mentally draining but emotionally very fulfilling and enriching for me. So, when I see my senior being helped or engaged or happy I feel a sense of fulfilment." (#14, S)*
Inner Setting	Readiness for Implementation (Available Resources)	Amount or level of available resources including funding, training, space and facilities available or manpower that allows the AAC staff the achieve the "AAC model" or the KPIs associated with it.	Lack of Manpower (B)	*"You know, we don't have enough manpower to reach out to all of them, basically. So, that would be an issue" (#10, CM)*<br>*"I mean, of course, three [manpower] is already not a lot. So now short (of) one is tough. So, we work on very lean resources, three head counts including myself." (#11, CM)*
			Space Constraints (B)	*"…in the morning, sometimes you see the whole bunch of seniors outside, because in here is so small, we cannot fit so many [seniors]. But when we turn them away that means they may never come back." (#1, CM)*<br>*"The space is very limited in terms of how many seniors we can accommodate." (#19, S)*
			Sufficient Training for staff (F)	*"So far all [courses] are very related, like the one I went through is [name of course]. It's very important for AAC staff… … very related to our job, so they are very beneficial." (#5, CM)*<br>*"For anyone who joins, it's [training] helpful because it helps you with operations. Training is great also because, it just equips you with the kind of skills that you require at an AAC." (#10, CM)*
			Time constraints for AAC staff (B)	*"In a sense, hey, like that I just [got to] do paperwork, just be a secretary and an administrator. Then how I go and serve seniors right? That part of it is very, very challenging because not much time on my hands after that already. Because apart from that, I still need to set up meetings to meet community partners... Then, yeah, no time for my seniors." (#9, CM)*

			Empowering seniors as a resource (F)	*"Okay one of the pool [of volunteers] is within the… amongst the seniors so we do identify some seniors among our seniors who are able to help. You know the lady who just sits there, she's a volunteer. She lives alone so she said "Anyway, I have nothing to do so I just come down and help." Really thankful for her. She comes down everyday." (#14, S)*
	Culture	Norms and values of the employees of an "AAC" in relationship to the elderlies (clients) and each other.	Culture of teamwork among staff (F)	*"I think I'm very blessed to have a very good team. So, our team has very strong teamwork. … … So, we are able to cover each other very easily." (#19, S)*<br>*"I like to think all of us are very communicative. We are quite open to communicate, and we have no issues covering for each other. I think we are quite a cohesive team in that sense." (#9, CM)*
	Network and Communications	The nature, quality and effectiveness of social networks between employees within the AAC or organization.	Availability of internal resource (F)	*"Our division, our team is actually very fortunate because we have combined home care service, home health service, elderly services and community case management into one division … … we are fortunate to have these resources which we leverage on." (#2, CM)*<br>*"And whenever I need help, the management is there to assist. "(#5, CM)*
	Physical Characteristics (open coded)	Physical characteristics of the AAC such as the physical appearance or physical location of the AAC itself.	Getting to centre is inconvenient for seniors (B)	*"Because the way ours is, is that we are not in the center of the whole thing[boundary], and we are at the far end of the whole area we've been given. From one end to the other, it takes is a three-bus stop ride." (#1, CM)*<br>*"Yeah, there are stairs. Yeah, there are stairs out of here. You come to here, you climb to third floor, third floor of the carpark. Then you're level with us." (#12, S)*
Outer Setting	Cosmopolitan	Degree of involvement of AACs with external organisations such as other AACs, governmental	Working with community partners (F&B)	*"Yeah, we are trying to work with the RC (Residential committee) over here, but seems to have some problems, it's a challenge working with RC. I've also tried to link with the community centre nearby. It has also been a challenge." (#7, S)*

		organisations and community partners.		*"I do work very closely with other community partners like our RC (residential committees), CC (community centres) and then [name of kindergarten]." (#11, CM)*
			Lack of sharing practices between AACs (B)	*"I mean I would like to know (how the nearby AACs are coping) … [It's] good to know where we are standing in terms of having a yardstick to know how my AAC is doing. Right now, I don't know how my surrounding AAC are coping." (#9, CM)*<br>*"Not really. One time only lor, networking session… … We did talk about setting up a group chat, you know, so we can share you have any programme, activity or if have any, you know we can… But didn't. Yeah didn't." (#11, CM)*
	External Policy & incentives	Governmental organisations' policy and regulations, mandates, recommendations and guidelines, pay-for performance, collaboratives and public or benchmark reporting.	KPIs affecting relationship with seniors (B)	*"It pushes us towards transactional relationship building, because you need to keep on hitting numbers, your focus is there, instead of that social capital building that you need to hold dear. Because old folks don't trust that easy right?" (#2, CM)*<br>*"I don't know whether this being in AAC actually helps the seniors or not. Because if you ask me for, from their [funding agency] point of view, maybe they just want to hit all these targets. But [we] outreach to them without really spending time with them to understand them more. So, I don't agree on this, if you ask me… Now, we don't even have time to go and visit the same seniors because we really have to do a different thing all the time." (#18, S)*
			Regular changes to KPIs (B)	*"Sometimes it [KPIs] changes quite rapidly….and it's not that easy on us because as operators for AACs, it takes time to do some implementation or through some transition or change… the KPIs need to be really on the ground up. I feel it's top down, but maybe [funding agency] people should come to the ground, work with the AAC people, understand the nature, the difficulty, the challenges before they build the policy and regulations on KPI upwards" (#7, S)*

				"Within the last four years, there's already five changes or amendments to the KPIs. So, we started with "ABC" [referring to the aims of the AAC model] then second year is still ABC but they expect us to "up tier". Then in the third year, they tell us of "ABC+2S" that was announced to us in July…So, the KPI keep changing. When the team is fully adjusted, the very next moment, the KPI change. So, it's a retraining, readaptation and the scope keeps widening. But it's definitely challenging because the skillset needed to perform the expected scope or spectrums of things becomes more complex." (#2, CM)
			Lack of clarity and explanations behind policies (B)	*"Then I think they [funding agency] [has] a guidebook and they [will] just update. Every now and then, they [funding agency] will update because they have the new KPI. But the rationale behind why it's changed I don't think…I mean at least for me, [it is] not explained." (#14, S)*
			Insufficient understanding of ground situation & limited guidance on achieving expectations (B)	*"I also get quite confused. Like if [funding agency] try to set this, but have they ever tried to be on the ground with us to see how difficult it is to just get the frail seniors to come?" (#18, S)*<br>*"I always say, [if] we cannot, we cannot do [achieve KPIs] right? Why don't you [funding agency] show us the KPI that you can do[achieve]. If you think it is manageable, can you show us how you'll do it? We will learn from you. You can show us how you do it, then we will know this is how you do it." (#16, CM)*
			Top-down approach for policies (B)	*"One thing is that the KPI need to be on, really on the ground up and not top down. You know in most of the regulations, I feel it's like top down but maybe [funding agency] people should come to the ground, work with the AAC people, understand, you know, the nature, the difficulty, the challenges before they build the policy and regulations on KPI upwards…So, this is something like [funding*

				agency] have to talk to us then they will probably understand better." (#7, S)
			Absence of referrals from primary healthcare (B)	*"So, we have been reaching out to the GPs [general practitioners] in the area. So, we have contact with like 2 GPs (general practitioners). So far, we have no referrals yet… …It's barely been a priority. Honestly, I think really because we have not had any health plans at all. I don't know why none of the seniors have come down with the health plans. I don't know if they are even on the healthierSG program, but we don't really see a lot of "2S" cases." (#10, CM)*<br>*"We never had referrals from there, from GPs (general practitioners) yet. … I think that the referral from the GPs is low because it's not their focus. None of the referrals came through." (#9, CM)*
	Patient Needs & Resources	Extent to which elderlies' needs are understood and prioritized by the AACs.	Importance of holistically addressing the social, physical and mental needs of the seniors (F)	*"I think actually, [the] active ageing concept is to make sure that the seniors come out, enjoy, you know, not only physically but [also] mentally. As you can say, age gracefully." (#7, S)*<br>*"I think that is something important, active ageing, uh for the senior's well-being in terms of physically and I mean active ageing includes social as well." (#14, S)*
	Public Perception on intervention (open coded)	A general perception of AACs among the general public, other stakeholders who may or may not be involved in the running of AACs.	Social stigma against AACs (B)	*"Those who are well and healthy, able, be it financial, be it mental, be it physical, perhaps they don't see the point to come down. "I'm well". And those who are well, they feel like maybe I have my own clique. Our branding has been stereotyped under the low-income group" (#3, CM)*<br>*"Because people, we hear they think that this centre is for poor people, or people who are not well off or with financial problems." (#7, S)*

Process	Engaging	The engagement of policy makers (AIC) to ground staff implementing the model (including staff and centre managers) which includes education, role modelling, training and others.	Ineffective engagement and feedback (B)	*"We will try to feedback to HQ [CCO headquarters] and see what HQ can feedback to [funding agency]. And certain things, I think, sometimes, [funding agency] also do understand, they do ask [for] our feedback, but we tell them the feedback, but they never come back with our feedback." (#7, S)* *"If you don't know where we are landing at, we can't make the necessary adjustments to arrive. So where, how and what? So those are not complete. I think if we have a clear direction and what is the end state we are looking at, rather than just number, number, number." (#2, CM)*
			Funding agency use of ambassadors (F)	*"With the [name of team] team, I think we have closed up the gaps. So, for anything we just go to our [name of team] representative who works very closely with us on the ground. I think that's a good thing, appreciative about that." (#11, CM)* *"I mean [funding agency] doesn't come here frequently. Okay so maybe come here only once a year or when you are doing certain projects, pilot projects then they will come. Sometimes uh I mean [funding agency] also have their team to come and share with us or ask us, to guide us you know certain things okay, but sometimes I felt that uh need to be constructive, don't be so directive" (#7, S)*

Abbreviations: CM- centre manager; S-staff; F- facilitator; B-barrier
