# Supplementary material for: Aging in place: implementation facilitators, barriers, and strategies in population health-oriented active aging centers in Singapore
Source: Front Public Health. 2026 May 4;14:1748178. doi: 10.3389/fpubh.2026.1748178 (PMC13180892; doi:10.3389/fpubh.2026.1748178)
Supplement: Supplementary file 2 [file Table_2.pdf]

Table S2. Implementation strategies adopted by AACs

CFIR domain/ constructs (Barrier/ facilitator identified)	Strategy used	Description of strategy	Representative quotes
Characteristics of individuals/ Traits of beneficiaries (Difficulty in attracting seniors to attend programmes - males)	Male-only activities	Some AACs identified activities for which male seniors are the only ones allowed to participant in an attempt to attract male seniors.	*"Actually, I started the tea appreciation is to target uncles [slang for male seniors]. I invite but nobody wanted to come there in the end all the aunties [slang for female seniors]. All the auntie, what to do with them? So, then they start asking me, why can't I join, why can't I drink tea?" (#11, CM)*<br>*"Then we have all male drink coffee ["lim kopi"] activity. So, we have that once a month only, a lot of places (other AACs) have it every week, but we have it once a month because ours was 30 over percent engagement of male seniors. They come here, they're very happy..." (#1, CM)*
Characteristics of individuals/ Traits of beneficiaries (Difficulty in attracting seniors to attend programmes – ethnic minorities)	Activities targeted to ethnic minorities	Some AACs have identified the ethnic minorities as a difficulty group to attract and have tailored programme.	*"So, we did try to get a Malay karaoke… … But maybe they come out for one session and then after that they drop out. I don't know why, they just don't feel the bind yet. I'm not sure. It is something to be studied." (#10, CM)*<br>*"We do this yearly like before (Hari) Raya, like the fasting month. Then, they will distribute porridge, and they will also have the, I mean, they collaborate with the mosque people. They will maybe give free stuff like pantry food all this, so they will come down… … So, there were like 200 plus over people." (#18, S)*
Inner setting/ Readiness for Implementation – available resources (Space Constraints)	Use of community resources for additional space	Many AACs have made use of spaces from other community partners	*"So [funding agency] have been asking us to work with our partners nearby okay so there okay this is a market okay there's a RC [resident's committee] here and then there's a RC there. Okay, we have contacted both and we are working with them both. Okay their RCs generally on weekdays are quite unused so we do run weekly programs here and here we have a bi-weekly uh Korean food here." (#15, CM)*
Inner setting/ Readiness for Implementation –	Empowerment of seniors	Some AACs have identified empowering seniors as a priority and hence some AACs rolled out	*"We have this programme called microjobbers. So, we empower our seniors to take on mini jobs, to which we will give them a small remuneration, but also ease the*

available resources (Lack of manpower & Empowering seniors as a resource)		the "micro-jobber" scheme, where seniors will be paid a fee for an ad-hoc "job". Other AACs who are not in this "micro-jobber" scheme provide training to train volunteers to assist staff with simple tasks.	*load on our AAC operations, as well as giving them a purpose when they are running their own activities." (#10, CM)*<br>*"Okay one of the pools [of volunteers] is within the… amongst the seniors so we do identify some seniors among our seniors who are able to help. You know the lady who just sits there, she's a volunteer. She… she lives alone so she said "Anyway, I have nothing to do so I just come down and help." Really thankful for her. She comes down everyday." (#14, S)*
Inner Setting/ Readiness for Implementation – available resources (Lack of manpower & Empowering seniors as a resource)	Self-sufficient running of activities	Considering the difficult targets set for the AACs, some AAC implemented a system whereby activities are run self-sufficiently by volunteers and whereby staff would strive towards the targets implemented.	*"I have volunteers running them (meaning activities) on autopilot mode already. So, most of them, most of the time, we do have regular volunteers… We have very good regular volunteers. If we don't have volunteers, then staff will need to come in to take over… We can free up the staff to go and chase the KPIs and do the other KPIs. But then again, so when you let everything autopilot by volunteers, you need to have to supervise the quality of it [their work], because not all volunteers are as good in a sense." (#9, CM)*
Inner Setting/ Culture (Culture of teamwork among staff)	Specialisation among staff	AACs splitting the job among staff due to the large number of requirements from funding agency	*"So down here [at this AAC], we are quite clear with our roles. We have a manager, CME is case management executive, VME is management… volunteer management executive. So, WC, wellness coach, that's my role. So, we are quite clear. But of course, sometimes we cross [do each other's roles]. … So, when I [conduct] exercise[s], so they [seniors] may have problems that I need the case people to [see]… We are clear with our roles, but we work together as a team." (#20, S)*
Characteristics of individuals/ Traits of beneficiaries (Difficulty in attracting seniors to attend programmes – "young" seniors")	Thematic Approach for AACs / Unique programmes	The thematic approach to AACs whereby different AACs have their own unique theme with specialised programmes according to this theme, providing seniors with a unique experience with programmes that are not usually offered by AACs.	*"You want to make it like a club style that "wow, your centre is like that." So, we are really looking into the thematic, as in the programmes that we run which is different. Like you see our [thematic programme], that's something [that is] very unique." (#18, CM)*<br>*"I think a lot of things here, thankfully, just comes forth, you know, initiated [from the] ground up… initiative by the seniors. … I mean, the ukulele group about started with the instructor, you know, who come and teach, and then, over the years, they*

			pick up the skills, and then now they can play quite confidently. Because of this dynamic, it [has] become an interest group …" (#11, CM)
External setting/ Cosmopolitan (Working with community partners)	Uniting community resources in a common forum	Having a political figure or central figure to bring all community partners together in the same locality and to work towards better services provided for seniors	*"Of course, we are seated in the [name of intervention] …So she [political figure] brings all the community partners together and she wanted to deconflict a lot of the services and programs by the different partners but for the same group of seniors in her constituency. This is pretty unique and that's the efforts of [political figure] at that level to bring the partners together." (#9, CM)*
